# Evaluation of unmet clinical needs in prophylaxis and treatment of venous thromboembolism in high-risk patient groups: cancer and critically ill

**DOI:** 10.1186/s12959-019-0196-6

**Published:** 2019-04-15

**Authors:** Benjamin Brenner, Russell Hull, Roopen Arya, Jan Beyer-Westendorf, James Douketis, Ismail Elalamy, Davide Imberti, Zhenguo Zhai

**Affiliations:** 1 Department of Hematology and Bone Marrow Transplantation, Rambam Health Care Campus, Haifa, Israel; 2 Foothills Medical Centre and Thrombosis Research Unit, University of Calgary, Calgary, Canada; 3 King’s Thrombosis Centre, Department of Haematological Medicine, King’s College Hospital NHS Foundation Trust, London, UK; 4 Thrombosis Research Unit, Department of Medicine I, Division Hematology, University Hospital ‘Carl Gustav Carus’ Dresden, Dresden, Germany; 5 Department of Medicine, McMaster University, Hamilton, Ontario Canada; 6 Thrombosis and Atherosclerosis Research Institute, Hamilton, Ontario Canada; 7 Hematology and Thrombosis Center, Tenon University Hospital, Sorbonne University, Paris, France; 8 Haemostasis and Thrombosis Center, Hospital of Piacenza, Piacenza, Italy; 9 Department of Pulmonary and Critical Care Medicine, Center of Respiratory Medicine, China-Japan Friendship Hospital, National Clinical Research Center for Respiratory Diseases, Beijing, China

**Keywords:** Venous thromboembolism, Cancer, Critically ill, Anticoagulants, Low-molecular-weight heparin

## Abstract

**Background:**

Clinical practice shows that venous thromboembolism (VTE) presents a substantial burden in medical patients, and awareness and advocacy for its primary and secondary prevention remains inadequate. Specific patient populations, such as those with cancer and the critically ill, show elevated risk for VTE, bleeding or both, and significant gaps in VTE prophylaxis and treatment exist in these groups.

**Objective:**

To present novel insights and consolidated evidence collected from experts, clinical practice guidelines and original studies on the unmet needs in thromboprophylaxis, and on the treatment of VTE in two high-risk patient groups: patients with cancer and the critically ill.

**Methodology:**

To identify specific unmet needs in the management of VTE, a methodology was designed and implemented that assessed gaps in prophylaxis and treatment of VTE through interviews with 44 experts in the field of thrombosis and haemostasis, and through a review of current guidelines and seminal studies to substantiate the insights provided by the experts. The research findings were then analysed, discussed and consolidated by a multidisciplinary group of experts.

**Results:**

The gap analysis methodology identified shortcomings in the VTE risk assessment tools, patient stratification approaches for prophylaxis, and the suboptimal use of anticoagulants for primary prophylaxis and treatment.

**Conclusions:**

Specifically, patients with cancer need better VTE risk assessment tools to tailor primary thromboprophylaxis to tumour types and disease stages, and the potential for drug–drug interactions needs to be considered. In critically ill patients, unfractionated heparin is not advised as a first-line treatment option, and the strength of evidence is increasing for direct oral anticoagulants as a treatment option over low-molecular-weight heparins.

**Electronic supplementary material:**

The online version of this article (10.1186/s12959-019-0196-6) contains supplementary material, which is available to authorized users.

## Background

Venous thromboembolism (VTE), which comprises deep vein thrombosis (DVT) and pulmonary embolism (PE), is a complex, multifaceted disease in which the clinical management in patients with cancer and the critically ill is challenging. Such patients are typically at increased risk for thrombosis and/or bleeding complications, and require special consideration for the prevention and treatment of VTE [1–3].

Despite the development of methodologically rigorous clinical practice guidelines that inform the prevention and treatment of VTE, adherence to such practices by healthcare professionals (HCP) is suboptimal. For example, although VTE-associated mortality has decreased considerably in surgical patients who receive postoperative thromboprophylaxis, in the high-risk patient groups identified above, thromboprophylaxis is commonly suboptimal and mortality rates remain high, with thrombosis being the second major cause of death in patients with cancer [4–7]. Moreover, overall survival of patients with cancer and VTE is shorter than those without VTE, even when accounting for tumour stage and cancer treatment [6, 7], and in critically ill patients, between 7 and 27% of deaths are due to PE [8].

Recent large, population-based studies show that anticoagulants are misused in terms of the appropriateness of the agent and dosing regimens administered [9]. In addition, despite the availability of risk stratification models for VTE and bleeding, they are complex, not optimally used in clinical practice and most require external validation [10, 11]. The alternative approach of systematically administering thromboprophylaxis to all at-risk medical patients in different clinical situations, for example in a post-hospitalisation period, without proper evaluation of VTE and bleeding risks could lead to unnecessary safety issues and has cost implications for healthcare systems [12].

Reduction of VTE incidence in medical patients is important to mitigate the risk of both initial VTE and VTE-associated sequelae comprising post-thrombotic syndrome [13] and chronic thromboembolic pulmonary hypertension [14]. In addition, VTE is associated with higher hospitalisation rates and longer in-hospital periods [15], resulting in a significant increase in the utilisation of healthcare resources.

Non-adherence to recommendations and guidance, or relying on expert opinion, remain important obstacles to the optimal administration of thromboprophylaxis [5]. Discrepancies between guideline recommendations, due to insufficient scientific evidence [16, 17], and differences in expert opinions highlight the need to review published evidence and clinical insights in order to identify unmet needs in prophylaxis and treatment of VTE, and potential ways to bridge these gaps between existing knowledge and practice.

## Main text

### Gap analysis methodology

The gap analysis methodology implemented involved qualitative research via telephone interviews, which took place from February to August 2017, with 44 experts from 12 countries or regions (Germany, France, Italy, Spain, UK, Brazil, Canada, China, Russia, Japan, Middle East and Africa). Using a pre-designed questionnaire [see Additional file 1], information was collected on prophylaxis and treatment of VTE in certain high-risk patient populations that were identified as those in which robust evidence and related practice guidelines were lacking or had considerable limitations. For the purpose of this paper, consolidated evidence collected from the interviews on gaps in the management of cancer and critically ill patients with VTE was supplemented through comprehensive quantitative research into published articles in PubMed and Cochrane Library from 2015 to 2017, and current guidelines published from 2015 until October 2018. Search terms included “cancer” or “tumour”, “acutely ill” or “critically ill”, and “venous thromboembolism”. Literature searches were supplemented with identification of seminal studies in the field and relevant systematic reviews published at any time on VTE in patients with cancer or the critically ill. Evaluation of research findings and further insights were obtained through author discussions at the Thrombosis Think Tank [Paris, 28 February 2018] meeting, and the information gained on these two patient populations, cancer and critically ill, are reviewed in this paper.

### Prevention and management of VTE in cancer

Despite an increase in the incidence of cancer in an ageing population around the world, survival of cancer patients is improving due to the introduction of novel therapies, improvements in existing treatment strategies and cancer prevention programmes. The annual incidence of VTE in patients with cancer ranges from 0.5 to 20% depending on the type of cancer, disease stage and associated treatment regimens, and is predicted to increase in the future [18], while VTE incidence in the general population remains unchanged [19]. Moreover, the risk of VTE is higher in more advanced stages of cancer, or with a metastatic disease, compared with less advanced cancer [20]. The association between VTE and cancer is also bidirectional, with 20% of all patients with VTE diagnosed with cancer, typically at the time that, or soon after, VTE is diagnosed [21–24]. The mortality rate in patients with cancer and concurrent VTE is 3-fold higher than that in cancer patients without VTE, and VTE is the second most common cause of death, after the malignancy itself, in patients with cancer [25]. Therefore, the primary and secondary prevention of VTE presents an important unmet need in this patient population.

#### Epidemiology of VTE in patients with cancer

Current guidelines highlight lung, pancreatic, ovarian, gastrointestinal and brain tumours, as well as haematological malignancies, as those associated with the highest risk of thrombosis; lymphoid, gynaecological (other than ovarian) and bladder tumours as those carrying high risk of thrombosis; and breast, head and neck, and prostate cancers presenting with a lower risk of VTE [18, 26–28].

The incidence of VTE varies not only according to the type of cancer, but also within cancer types, specifically lung, ovarian, oesophageal, gastric, pancreatic and brain cancers, and chronic lymphocytic leukaemia. The incidence of VTE is particularly high in lung, pancreatic and brain cancers [29–31], and a paucity of data on how to manage these patients was noted by the experts who were interviewed, especially when patients are undergoing chemotherapy. For example, in a study of patients with lung cancer who received cancer treatment with curative intent, the cumulative incidence of VTE after 1 year reached 13.5% [29], while another study that examined VTE incidence in patients with non-small-cell lung cancer reported 6-month and 2-year rates of VTE of 4.2 and 6.4%, respectively [32]. In general, VTE incidence in lung cancer varies widely, from 1.3 to 21.5% [33–36], but the presence of VTE is consistently associated with a worse overall survival in such patients [36–38].

A systematic review and meta-analysis of VTE incidence rates reported a 2-year cumulative incidence rate of 11.2% in advanced pancreatic cancer [30], while in a more recent study the incidence of symptomatic and asymptomatic VTE was 16.5% in patients with pancreatic cancer [39]. In a meta-analysis of patients with brain tumours, the risk of VTE was found to be significantly related to glioma (risk ratio [RR] = 1.68, *P* < 0.001), high-grade glioma (RR = 1.70, *P* < 0.001) and glioblastoma multiforme (GBM) (RR = 1.74, *P* < 0.001) [31]. Overall data on risk for VTE according to the type of cancer and staging will guide clinical decisions on the use of thromboprophylaxis.

Clinical practice guidelines provide evidence-based recommendations for the primary prevention of VTE in patients with multiple myeloma (MM) who are undergoing treatment with cytotoxic chemotherapy and immunomodulatory imide drugs (IMiD) [40]. This evidence is based on the association of MM with VTE. At baseline the VTE rate is 3–4% in these patients, and this is further increased with exposure to risk factors, including treatment with high-dose dexamethasone, cytotoxic chemotherapy (doxorubicin), IMiDs (thalidomide and lenalidomide), erythropoiesis-stimulating agents, reduced mobility, fractures, and personal or family history of thrombosis [40, 41].

Chemotherapeutic regimens also contribute to the development of VTE, as the annual incidence of VTE in cancer patients treated with chemotherapy is 1 in 200, while in the general population it is 1 in 855 [42]. Outpatients receiving chemotherapy have a 6.5-fold increase in the risk of VTE compared to patients not treated with cytotoxic therapy [43], and the second most common cause of death in patients receiving outpatient chemotherapy is VTE [44].

In summary, expert opinion and a number of studies highlight the importance of identifying high-risk patients within cancer populations, who may benefit from thromboprophylaxis to prevent VTE and, in turn, decrease VTE-associated morbidity and mortality.

#### Risk assessment models and biomarkers for prediction of primary and recurrent VTE in patients with cancer

Guidelines from the British Committee for Standards in Haematology [45] recommend the Khorana risk score (KS) as a tool for categorising patients into very high VTE risk patients, such as those with gastric and pancreatic cancer, and high VTE risk patients, such as those with lung, gynaecological, bladder or testicular cancer, or lymphoma [45]. However, more recent evidence has shown that a high-risk score does not necessarily predict presence of VTE in patients with lung cancer, although it does associate with all-cause mortality [46]. Moreover, the KS has insufficient precision in stratifying patients with lung cancer who are receiving chemotherapy into high- and low-risk groups [47], and those with pancreatic cancer into high- and intermediate-risk groups [48, 49]. The latter high-risk patient group, however, could be identified by combining the KS or CONKO scores with an activated partial thromboplastin time [48]. In addition, the predictive value of the KS in cancer-associated thrombosis was higher when the analysis included platinum-based chemotherapy and the presence of distant metastases [50]. Another VTE risk assessment tool, the Ottawa score, considers the type of cancer, disease stage, gender and history of thrombosis, but it could not adequately predict recurrent VTE in patients receiving anticoagulants [51]. The risk of VTE fluctuates throughout a patient’s disease course, and the type of cancer, stage and therapeutic regimens will have an impact on the level of VTE risk [52]. The experts recognise that there is an unmet need for improving VTE risk assessment tools for identifying cancer patients at risk, and for balancing those risks against anticoagulant-induced bleeding.

Current risk-assessment-based decisions that usually rely on clinical parameters as potential VTE biomarkers, including D-dimer and other biomarkers, are unlikely to have a significant impact on routine clinical practice in the foreseeable future [25]. However, a recent systematic review on biomarkers for prediction of thromboembolism in lung cancer demonstrated that D-dimer and epidermal growth factor receptor mutation were the most reproducible predictors of thromboembolism in this patient population [53]. Circulating tissue factor emerged as a potential biomarker in its highest quartile, where it was associated with the highest VTE recurrence rate in patients with cancer who were receiving anticoagulants, but this marker is not widely used in clinical practice [54].

Taken together, and with the experts advocating for personalised treatment based on risk-assessment models, these data demonstrate an urgent need to develop practical, realistic and useful risk assessment tools, which would be able to stratify cancer patients into high-, intermediate- and low-risk primary and recurrent VTE groups eligible for targeted thromboprophylaxis approaches.

#### Primary prevention of VTE in patients with cancer

Primary prophylaxis in patients with cancer should be considered, as the experts agreed on the importance of improving prevention of VTE, which presents a challenge in terms of recurrent thrombosis and clinically relevant bleeding. It was acknowledged that significant improvements in inpatient thromboprophylaxis have occurred over recent years; however, beyond hospitalisation the benefits of extended prophylaxis remain less well-defined and require further investigation as patients may remain at risk of VTE after hospital discharge. Current guidelines advise against routine thromboprophylaxis with low-molecular-weight heparin (LMWH) in ambulatory patients with active cancer receiving systemic anticancer therapy, such as adjuvant hormonal therapies or chemotherapy [45, 55, 56]. Primary prophylaxis may be indicated in specific subpopulations only, including those with pancreatic or lung cancer, ambulatory patients at high thrombotic risk [12, 27] and those receiving chemotherapy for prolonged periods of time [57]. However, antithrombotic therapy that extends beyond 6 months is controversial, but may be advised for patients with metastatic disease receiving chemotherapy, immunochemotherapy or radiotherapy [58]. It is also recommended that patients with cancer and reduced mobility who are admitted to hospital, or who are treated with thalidomide and lenalidomide combined with steroids or other systemic anticancer therapies, should receive prophylaxis with LMWH, unfractionated heparin (UFH) or fondaparinux [7, 59]. In at-risk patients, VTE prophylaxis should begin as soon as VTE risk has been identified [12], but administration of anticoagulants should consider comorbidities associated with cancer, the risk of bleeding and patient preferences [27].

In patients with metastatic disease and high risk of bleeding, LMWH is the preferred option over other anticoagulants, while vitamin K antagonist (VKA) should be avoided [6, 55]. However, in patients with renal failure, LMWH is not routinely recommended, as it increases the risk of major bleeding [57]. In patients with MM at high risk of VTE, full-dose LMWH or adjusted-dose warfarin (targeted international normalised ratio ∼1.5) are recommended, in contrast to MM patients with low-risk factors who are advised low-dose aspirin [7, 57]. A recent systematic review on MM patients who were treated with lenalidomide-based therapy and/or dexamethasone showed a reduction in VTE risk for patients on LMWH (1.4%) compared to patients on aspirin (10.7%) [60].

Clinical practice guidelines recommend LMWH for thromboprophylaxis in low-bleeding-risk patients with locally advanced or metastatic pancreatic cancer or lung cancer treated with systemic anticancer therapy [7]. The benefits of LMWH in reducing VTE risk were also demonstrated in the CONKO-004 trial, where the rates of symptomatic VTE reduced by 8.7% in patients with advanced-stage pancreatic cancer receiving primary prophylaxis with enoxaparin, compared to patients not receiving prophylaxis [61]. Fondaparinux should be considered in patients with previous heparin-induced thrombocytopenia (HIT) [6]. The role of direct oral anticoagulants (DOAC) for the prevention of VTE in cancer patients is uncertain [27]. Recent results from the CASSINI trial demonstrated the safety and efficacy of thromboprophylaxis with rivaroxaban in patients with cancer [62]. The study examined this DOAC for VTE prevention in ambulatory patients with various cancers and found that VTE and VTE-related deaths were significantly reduced during the on-treatment period, and major bleeding was low. However, the AVERT trial, which examined the safety and efficacy of apixaban to prevent VTE in high-risk cancer patients, found that although lower rates of VTE were observed in the apixaban group compared with the placebo group, major bleeding rates were much higher [63]. These contradictory results suggest that more trials are needed to consolidate future recommendations on the use of DOACs in this patient population [62].

#### Secondary prevention and treatment of VTE

In cancer patients who develop recurrent VTE despite appropriate anticoagulant therapy, expert opinion guidance suggests three treatment options, which include increasing the LMWH dose by 20–25%, switching therapy from VKA to LMWH, or inserting an inferior vena cava (IVC) filter in combination with anticoagulation therapy [7]. If a patient with cancer develops DVT or PE, treatment guidelines recommend initiating treatment with a once-daily LMWH regimen, with suggested pharmacological alternatives of fondaparinux for patients with ongoing or prior HIT, or UFH for patients with severe renal insufficiency or who are dialysis-dependent. An IVC filter should only be considered in selected patients with an absolute contraindication to anticoagulant therapy, given the prothrombotic stimulus of such foreign bodies [6, 7]. Finally, thrombolytic therapy should only be considered in patients with clinically massive DVT or PE, and with caution given the increased bleeding risk of patients with cancer [7]. A minimum of 3 months’ anticoagulant therapy is recommended, with LMWH preferred over VKAs and with consideration given to at least 6 months of treatment, especially in patients who are receiving cancer treatment or with metastatic disease [7, 59, 64, 65]. However, the qualitative interviews highlighted a lack of consensus among physicians regarding treatment after the initial 6-month period. Indeed, the DALTECAN study examined the efficacy and safety of up to 12 months’ treatment with dalteparin, and found that VTE recurrence and bleeding were clustered during the initial month after diagnosis, thereby supporting the long-term safety of LMWH therapy [66]. After 6 months’ treatment, the need for ongoing anticoagulant therapy should be reassessed based on a risk versus benefit assessment in conjunction with patient values and preference [12].

The lack of published data on the benefits of VTE reduction through thromboprophylaxis versus the risks of bleeding was highlighted by the experts as one of the reasons why guideline recommendations are not routinely followed by physicians. Patients with GBM treated with lifelong anticoagulation have a reduced rate of recurrent VTE but, despite these findings, thromboprophylaxis is underutilised in such patients [67]. According to the experts interviewed, this may be partly due to differences in the views of oncology specialists regarding the need for treatment of established VTE compared to the role of primary thromboprophylaxis. In contrast, another study demonstrated that patients with cancer treated with anticoagulant therapy suffered a 3-fold higher incidence of intracranial haemorrhage [68]. The differences in the rate of VTE recurrence incidence and major bleeding events were linked to the type of cancer, as a study demonstrated that the rates of VTE recurrence and major bleeding events during the course of anticoagulation were similar in patients with breast or colorectal cancer, whereas a 2-fold higher rate of thromboembolic recurrences than the rate of major bleeding events was identified in patients with lung cancer, and a lower rate of VTE than bleeding was recorded in patients with prostate cancer [19]. These data underscore the importance of considering the type of cancer and associated comorbidities in order to weigh the potential benefits and risks of anticoagulant prophylaxis.

For the past two decades, LMWHs have been the preferred first-line treatment option for the management of cancer-associated VTE. This is supported by results from the qualitative survey, which found that 75% (33/44) of physicians interviewed use LMWH as standard of care for thrombosis treatment. Several seminal studies have shown superior efficacy and safety of LMWHs over VKA. The CANTHANOX study reported 10.6% more patients experiencing one combined major outcome event, such as major bleeding or recurrent VTE within a 3-month period, in the warfarin group compared to the fixed-dose enoxaparin group [69]. The CLOT study demonstrated that a weight-adjusted dose of dalteparin was more effective in reducing the probability of recurrent VTE compared to a warfarin derivative over a 6-month treatment period [64]. Similarly, the LITE study found a greater number of VTE episodes in the VKA than the tinzaparin treatment group at 3 and 12 months, with largely similar minor bleeding complications [70], whereas the more recent CATCH study demonstrated a similar rate of recurrent VTE over a 6-month period with tinzaparin compared to warfarin, but a lower rate of relevant non-major bleeding was noted in the tinzaparin group [71]. The cumulative probability of being VTE-free at 6 months, as demonstrated by the ONCENOX study, was higher for the group receiving enoxaparin than for the group where treatment with enoxaparin preceded that with warfarin [72]. In summary, LMWHs seem to be preferred anticoagulants over VKAs.

More recently, DOACs have emerged as a potential alternative first-line treatment option for cancer-associated VTE, but with caveats. In general, the type of anticoagulant administered should be tailored according to patient and cancer type characteristics. The expert discussions and interviews highlighted that the advantages of LMWHs over DOACs include the ease of adapting the dose to the patient’s body weight and anticoagulation need, no drug–drug interactions related to chemotherapy regimens, and flexibility around procedures and other clinical situations (e.g., thrombocytopenia) that require treatment interruption or dose reduction. However, the experts agreed that prolonged drug administration through subcutaneous injection was the most common disadvantage associated with LMWH treatment, followed, in certain countries, by the relatively high cost of LMWH. Currently, DOACs are used for treatment of VTE in patients with stable cancer who are not receiving anticancer therapy and when VKAs are unavailable [59]. Some published studies, specifically the AMPLIFY trial [73] and the Hokusai-VTE trial [74], demonstrate that DOACs have similar efficacy to that of LMWHs or warfarin. Nevertheless, the authors agreed that bleeding risk associated with DOACs should be addressed, as bleeding may be a more frequent cause of death than fatal VTE. The HOKUSAI-CANCER study compared dalteparin with edoxaban over a 12-month period and demonstrated a comparable rate of VTE recurrence in both groups, although a higher rate of major bleeding was observed in the edoxaban group [75]. In addition, in the recent SELECT-D study, patients treated with rivaroxaban displayed a lower cumulative VTE recurrence rate than those treated with dalteparin, but had a higher rate of major and clinically relevant non-major bleeding events in the 6-month period [65].

The experts noted that the administration of LMWHs and DOACs may become interchangeable, as patients with cancer have a complex clinical course and receive many different therapies; for example, it is possible to envisage that LMWHs will be used during hospitalisation and DOACs in out-of-hospital periods. The cost was considered to be one of the major reasons for insufficient adherence to guideline recommendations for the use of LMWHs. Among other reasons for the suboptimal use of LMWHs are inconvenience of LMWH injections and insufficient awareness of care givers regarding the importance of secondary prevention of VTE.

In conclusion, it is important to consider cancer site, stage of the disease and anticancer treatments given to patients to ensure the choice of an optimal anticoagulant and its dosage for secondary prevention of VTE.

### Inadequate management of VTE in critically ill patients

VTE is a frequent cause of preventable morbidity and mortality in hospitalised acutely ill patients [76], and it is recognised that the main burden of disease occurs in an out-of-hospital setting when in-hospital thromboprophylaxis ceases upon discharge of these patients from hospital. DVT rates usually range from 13 to 31% in critically ill patients without thromboprophylaxis [77], and 26% of patients with undiagnosed or untreated PE will have a subsequent fatal embolic event [78, 79]. Moreover, a non-fatal recurrent embolic event will occur in another 26% of patients with PE [78, 79]. Continuous improvement in thromboprophylaxis in hospitalised acutely ill patients has occurred over the past decade, but expert consensus suggests prevention of VTE still remains a significant unmet need in medical patients compared to prevention of VTE and its management in surgical patients.

#### Stratification of VTE and bleeding risks in critically ill patients

At least one VTE risk factor is present in most hospitalised critically ill medical patients, and usually the risk of VTE remains in patients for several weeks after discharge from hospital [80]. Despite this problem, insufficient published data exist on the validity of risk scores for determining the risk level of VTE in critically ill patients. Some studies have investigated how to define subgroups of critically ill patients in which the benefit-to-risk ratio is in favour of thromboprophylaxis [81, 82]. The EXCLAIM study compared efficacy and safety of extended-duration thromboprophylaxis with enoxaparin to placebo in acutely ill medical patients with prolonged reduced mobility [81]. The study found that extended administration of enoxaparin reduced the rate of VTE incidence from 4 to 2.5% but increased the rate of major bleeding events (0.8% versus 0.3% favouring placebo). However, the benefit of reduction of VTE incidence in some acutely ill medical patient subgroups, including patients with level 1 immobility, patients > 75 years of age and women, outweighed the risk of increase in major bleeding events [81]. The MAGELLAN trial demonstrated that D-dimer is an independent predictor of VTE risk (odds ratio 2.29) and a 3.5-fold greater incidence of VTE was detected in patients with D-dimer concentrations > 2-fold higher than the upper limit of normal (ULN) baseline value [82]. However, the experts agreed that D-dimer has little value as a biomarker of VTE in clinical practice and it is currently used for a purely scientific purpose. Decisions on prophylaxis are usually based on clinical parameters. Implementation of D-dimer in routine clinical practice for critically ill patients as part of a risk stratification model is difficult, as significant variation between the assays and protocols in different hospitals exists, thus making standardisation of the assay problematic. The experts noted that D-dimer is not a good marker for VTE for patients in the intensive care unit (ICU), as other factors, such as infection, can affect D-dimer levels. However, if D-dimer levels increase over a stay in hospital, then it could be indicative of thrombosis.

Moreover, the qualitative interviews highlighted limitations in carrying out assays in certain countries where samples had to be sent abroad for testing, or where patients lived in out-of-reach rural areas where general practitioners are in need of simple stratification methods for identifying high-risk patients. Indeed, 58% (14/24) of experts questioned considered that improved estimation of an individual’s benefit-to-risk ratio required further experimental investigation to allow better patient stratification for personalised prophylactic anticoagulant therapy.

#### Prophylaxis and treatment regimens in critically ill patients

Thromboprophylaxis is recommended to all ICU patients, including high-risk patients with immobility until mobility is restored [83]. However, the experts agreed that there are no clear recommendations on how to identify patients at risk or on how to manage asymptomatic VTE, which is an underlying cause for symptomatic VTE. Moreover, in the recent APEX trial, acutely ill patients who were assessed for, and found to have, asymptomatic DVT had a 3-fold increased risk of short-term all-cause mortality [84].

LMWH is the anticoagulant of choice used in critical care units with a guideline-suggested enoxaparin dose of 40 mg once daily [12, 85]. The beneficial effects of enoxaparin given at 40 mg dose compared to placebo extend to a wide range of acutely ill medical patients, including patients with acute or chronic heart failure, acute or chronic respiratory failure, acute infectious disease, acute rheumatic disorder, cancer, a history of VTE or immobility [85]. The choice of a particular LMWH should be based on the magnitude of a clinical effect, level of evidence, approval by the regulatory authorities for each indication, and cost [79]. Consideration should be given as to whether LMWH should be administered once or twice per day depending on a specific clinical situation, while taking into account safety and a patient’s renal function. Dalteparin and tinzaparin have safety advantages compared to enoxaparin when renal impairment manifests, i.e., when creatinine clearance is below 30 mL/min, because of differences in molecular weight and the LMWH clearance pathway, therefore prophylactic doses of dalteparin and tinzaparin in patients with impaired renal function are safe [86]. In contrast, fondaparinux is currently contraindicated in critically ill patients with severe renal disease [87].

Evidence suggests that LMWH should be a preferred option over UFH in critical care units, as the latter is linked to HIT and requires more frequent dosing [88]. The study of dalteparin and UFH efficacy and safety has demonstrated that proximal leg DVT rates are similar in the dalteparin and UFH groups, but treatment with dalteparin results in a significantly lower proportion of patients with PE when compared to treatment with UFH, while the rates of major bleeding or death in the hospital are similar between the groups [89]. The Avoid-Heparin Initiative proved additional benefits of using LMWH over UFH by demonstrating the decrease of the annual rate of suspected HIT by 42% (*P* < 0.001) and the annual rate of patients with a positive HIT assay by 63% (*P* < 0.001), where adjudicated HIT and HIT and thrombosis decreased by 79% (*P* < 0.001) and 91% (*P* < 0.001), respectively [88]. Moreover, hospital HIT-related expenditures decreased by US$266,938 per year in the avoid-heparin phase [88].

The duration of anticoagulant therapy may be restricted by the presence of individual risk factors for anticoagulant-induced bleeding. Thrombosis Canada guidelines note that patients > 75 years of age, those with renal or liver failure, patients with previous stroke history and those with cancer are at a higher risk of bleeding [76]. The International Stroke Trial, a large study of 14,578 patients with suspected acute ischaemic stroke, demonstrated that treatment with heparin was associated with an increased risk of haemorrhagic or serious extracranial haemorrhagic stroke. However, this was outweighed by a decrease in recurrent ischaemic stroke [90] and the American College of Physicians recommended the use of heparin for VTE prophylaxis in stroke patients, provided evaluation of individual VTE and bleeding risks are carried out [79].

If pharmacological thromboprophylaxis is contraindicated due to high risk of bleeding, then application of elastic compression stockings or intermittent pneumatic compression is advised [91]. Mechanical compression is very effective in ICU patients and can be used in combination with pharmacological intervention [92], although the expert interviews suggest that compliance to mechanical compression is low in the ICU.

According to experts in countries where patients are expected to pay for medication, cost is a factor that restricts both prescription of prophylactic drugs and patient up-take of a prescribed medication. Prescriptions are limited to high-risk patients, those admitted to hospital for long periods or those undergoing surgical intervention. LMWH is available exclusively in special hospital units in these countries.

DOACs are not currently recommended by the guidelines for thromboprophylaxis in critically ill patients, and the authors agreed that LMWH is a preferred choice of an anticoagulant during hospitalisation, but after discharge from hospital a switch to DOACs may be recommended. In support of this statement, the APEX study demonstrated that a similar proportion of patients receiving extended thromboprophylaxis with betrixaban or standard-duration enoxaparin therapy achieved reduction in a composite of asymptomatic proximal DVT and symptomatic VTE without differences in frequency of major bleeding events [93]. Betrixaban also showed efficacy at an 80 mg dose versus standard-dose subcutaneous enoxaparin (40 mg once daily 10 ± 4 days) with a better primary efficacy outcome (6.27% versus 8.39%; relative risk reduction 0.26; *P* = 0.023). However, a greater risk of clinically relevant non-major bleeding compared to the enoxaparin group was observed [94]. The subpopulation analysis that involves patients with stroke also suggests benefits of betrixaban, as extended-duration treatment with betrixaban significantly reduced all-cause stroke and ischaemic stroke during a 77-day follow-up period [95]. The exploratory post-hoc analysis in patients with a history of VTE revealed that only 12 patients would need to be treated with betrixaban to prevent an additional VTE endpoint [96]. Extended thromboprophylaxis with betrixaban was more efficient compared with standard-duration enoxaparin, and resulted in reduced risk of VTE-related rehospitalisation at day 42 (0.25% versus 0.76%; hazard ratio [HR] 0.33) and at day 77 (0.46% versus 1.04%; HR 0.44) [96].

#### Extended prophylaxis beyond hospitalisation

According to our qualitative research, expert opinion on the benefits of extended prophylaxis is divided due to a lack of evidence and contradictory guidelines. The experts stressed that acutely ill patients are exposed to an elevated risk of VTE after discharge from hospital, as VTE prophylaxis remains inadequate during this period, thus having an adverse effect on patient outcomes and presenting a financial burden on the healthcare system [80]. However, a prospective observational study reported that out of 5.5% of hospitalised critically ill patients who developed VTE, 74.1% of patients acquired VTE during hospitalisation and 25.9% following discharge, and the latter was affected by a history of VTE, recent surgery and pulmonary conditions [80]. ADOPT, EXCLAIM, MAGELLAN and APEX studies involved acutely ill hospitalised patients, examining efficacy and safety of extended-duration thromboprophylaxis with either enoxaparin or DOACs, including apixaban, betrixaban or rivaroxaban [81, 93, 97, 98]. These studies showed that following a 30-day or longer treatment period (up to 35 ± 4 days), VTE rates range from 2.5 to 5.7%, suggesting that significant VTE burden occurs well beyond hospitalisation [81, 93, 97, 98]. The ADOPT trial demonstrated an immediate increase in VTE risk when standard-duration (6–14 days) prophylaxis with enoxaparin was stopped [97]. Considering that acutely ill hospitalised patients are usually discharged before 5 days of hospitalisation and prophylaxis is discontinued post-discharge, VTE risk remains high in this period [97]. Extended-duration prophylaxis (28 ± 4 days) with enoxaparin administered to both hospitalised patients and outpatients was effective in reducing VTE incidence from 4 to 2.5% according to the EXCLAIM study [81]. The MAGELLAN study showed that rivaroxaban administered for an extended period (35 ± 4 days) was superior to standard-duration (10 ± 4 days) enoxaparin [98]. However, both the EXCLAIM and MAGELLAN studies showed an increase in major bleeding risk. The sub-analysis of the MAGELLAN study revealed that the high D-dimer (> 2-fold the ULN baseline level) group responded equally as well to enoxaparin or rivaroxaban at day 10, but rivaroxaban was superior to placebo at day 35 [82]. It has been noted that testing at the end of a 10-day prophylaxis regimen may identify individuals at greater risk of VTE who would benefit from extended therapy, but the impracticalities of collecting samples for laboratory tests on or after discharge from hospital have also been highlighted [82]. The APEX study demonstrates the benefits of extended prophylaxis with betrixaban in critically ill patients, but due to reduced length of hospital stay becoming more common and the discontinuation of these drugs post-discharge, thromboprophylaxis may remain inadequate [93].

While the above studies highlight the benefits of extended prophylaxis using enoxaparin or DOACs beyond the standard in-hospital therapy, they did not directly compare VTE rates in patients who received anticoagulant therapy versus those who had no prophylaxis beyond the hospital stay. Recent results from the MARINER trial that directly evaluated the efficacy and safety of thromboprophylaxis – in this case with rivaroxaban, compared to a placebo – in preventing symptomatic VTE in high-risk medical patients from the start of hospital discharge to 45 days post-discharge found that extended thromboprophylaxis did not significantly lower the risk of VTE or death [99].

Based on findings from the APEX study, the US Food and Drug Administration (FDA) approved betrixaban for extended thromboprophylaxis in critically ill medical patients at risk of VTE due to moderate or severe restricted mobility [100]. However, this has not been the case in Europe where the Committee for Medicinal Products for Human Use found that on review of the data from APEX, the safety and efficacy of betrixaban was not robustly demonstrated [101]. In addition, recent guidelines published by the American Society of Hematology advise against extending pharmacological prophylaxis following hospital discharge [102]. Therefore, although post-discharge VTE prophylaxis may be warranted, a more liberal use of this concept outside of the selection criteria of APEX is not recommended.

## Conclusions

In comparison to surgical patients, where VTE management has improved considerably, thromboprophylaxis for critically ill and cancer patients remains inadequate. This is partly due to a lack of relevant clinical trials in these patient populations, but may also be linked to the complication of comorbidities and a lack of understanding of HCPs on how to balance prophylaxis and bleeding risk. Despite an increase in survival rate of cancer patients, VTE incidence remains at an elevated level and is one of the major causes of mortality in this patient population. High VTE risk is linked to certain chemotherapeutic regimens and cancer or tumour types, therefore, the ability to stratify patients according to these criteria would improve VTE outcomes in cancer patients. However, biomarkers and risk assessment scores are inconsistent and in both critically ill and cancer patients, tests such as D-dimer may be affected by other factors related to the patient’s illness, such as infection, rendering the test unreliable.

Due to its safety and efficacy profile, LMWH remains the first choice of prophylaxis in critically ill and cancer patients. VKA is not advised for patients with cancer and UFH is not recommended in critically ill patients due to the risk of HIT. The safety of DOACs due to the increased bleeding risk needs to be examined further before they can be used routinely in patients with cancer. However, a recent study and an FDA approval on the use of betrixaban for extended thromboprophylaxis in critically ill patients demonstrates the effectiveness of this DOAC in reducing VTE incidence.

Despite improvements in in-hospital thromboprophylaxis, post-discharge, the use of thromboprophylaxis needs to be clarified. In both critically ill and cancer patients, the risk of VTE extends outside the hospital stay, but out-of-hospital risk assessment is impractical and recent studies, FDA approval, European Medical Agency rejection and guideline recommendations on extended use of thromboprophylaxis following discharge are contradictory. Therefore, out-of-hospital thromboprophylaxis should be carefully considered according to a patient’s benefit-to-risk profile.

Future studies need to examine methods of stratifying patients with cancer according to stage of disease, cancer site and cancer treatment, to improve knowledge regarding anticoagulant regimens in order to decrease the risk of bleeding and VTE recurrence. In critically ill patients, the burden of VTE lies outside the hospital setting and therefore, more studies are needed to examine practical means of risk assessing patients after discharge and to compare thromboprophylaxis options considering patient preferences, comorbidities and bleeding risk.

## Additional file

Additional file 1: Qualitative interview questionnaire. (DOCX 16 kb)

## Abbreviations

DOAC: Direct oral anticoagulants
DVT: Deep vein thrombosis
FDA: US Food and Drug Administration
GBM: Glioblastoma multiforme
HCP: Healthcare professional
HIT: Heparin-induced thrombocytopenia
HR: Hazard ratio
ICU: Intensive care unit
IMiD: Immunomodulatory imide drugs
IVC: Inferior vena cava
KS: Khorana risk score
LMWH: Low-molecular-weight heparin
MM: Multiple myeloma
PE: Pulmonary embolism
RR: Risk ratio
UFH: Unfractionated heparin
ULN: Upper limit of normal.
VKA: Vitamin K antagonist
VTE: Venous thromboembolism
