# Supplementary material for: Evaluation of unmet clinical needs in prophylaxis and treatment of venous thromboembolism in high-risk patient groups: cancer and critically ill
Source: Thromb J. 2019 Apr 15;17:6. doi: 10.1186/s12959-019-0196-6 (PMC6466798; doi:10.1186/s12959-019-0196-6)
Supplement: Supplementary file 1 — Qualitative interview questionnaire. (DOCX 16 kb) [file 12959_2019_196_MOESM1_ESM.docx]

Additional file 1

*Qualitative interview questionnaire*

|  |
| --- |
| **Question:** |
| **PART ONE** |
| **Personal:** |
| What do you consider to be your clinical speciality? |
| What is your main area of interest relating to thrombosis and anticoagulant therapy? |
| **PART TWO** |
| **Extended medical prophylaxis in special standalone population and indications** |
| In which high-risk subpopulation(s) of patients, if any, do you think medical prophylaxis is not currently being used optimally, e.g., under prophylaxis or inadequate duration? |
| How do you stratify patients for extended medical prophylaxis and assess benefit to risk?  Which tools/scores do you use, if any? |
| Are the guidelines regarding use of prophylaxis in this/these population(s) clear or is there a need for more specific guidelines or local protocols? |
| Are there any economic barriers to optimal prophylaxis in this/these population(s)? |
| **PART TWO (a)** |
| **Patients with cancer at substantial risk of recurrent thrombosis** |
| Which anticoagulants are indicated by the current evidence base for treatment of cancer patients with recurrent thrombosis? |
| Are there any reasons why guideline recommendations for specific anticoagulants, such as LMWHs, in cancer patients with recurrent thrombosis are not followed? |
| What factors influence use of LMWHs such as enoxaparin for secondary prevention of thrombosis in cancer patients? |
| Are additional support tools or evidence needed to convince HCPs of the cost-effectiveness of enoxaparin? |
| Are there any other ways in which appropriate use of enoxaparin in cancer patients with recurrent thrombosis could be better defined? |
